# Promotion of Pregnant Merino Ewes’ Welfare with the Introduction of a Drought- and High-Temperature-Resistant Cereal into Their Diet: Analysis of Tritordeum Meadow

**DOI:** 10.3390/ani13193155

**Published:** 2023-10-09

**Authors:** Estrella I. Agüera, Lucía Requena, María B. García-Moreno, Manuel A. Pérez-Priego, Francisco Requena

**Affiliations:** 1 Cellular Biology, Physiology and Immunology Department, Faculty of Veterinary, University of Cordoba, Campus of Rabanales, 14071 Cordoba, Spain; ba1agbue@uco.es (E.I.A.); v92redol@uco.es (L.R.); v02redof@uco.es (F.R.); 2 Statistics and Econometrics Department, University of Cordoba, Agri-Food Campus of International Excellence ceiA3, 14071 Cordoba, Spain

**Keywords:** cereal, drought, ewes, functional food, grazing, physiological parameters

## Abstract

**Simple Summary:**

Nutrition is one of the strategies for improving animal welfare because it meets the metabolic, functional, and physical needs of animals. Tritordeum is a robust cereal with yields like different wheat varieties, with high resistance to drought, a high-temperature stress, pathogens, and low fertiliser requirements; these characteristics that make it suitable for its use in sustainable production systems with low environmental impact. In addition, it has specific properties and functionalities that are not found in any other cereal. Physiological indicators of animal welfare have been evaluated in line with the new regulatory guidelines of the European Union’s Common Agricultural Policy, making ovine farmers aware and respectful of animal welfare, considering strategies like the inclusion of this new cereal with beneficial health effects in their livestock diets.

**Abstract:**

Tritordeum is a new cereal resistant to drought and high temperatures, and it is a very healthy crop. The aim of this study was to compare two different diets (tritordeum meadow vs. oat meadow) for grazing pregnant ewes to determine if there was any effect on the objective physiological indicators of animal welfare. A total of 150 pregnant Merino ewes (3–5 years) were randomly divided into two groups (*n* = 75 each) to be fed with two different meadows, being evaluated during the spring season. Red blood cells count, haemoglobin, packed cell volume, white blood cell count, neutrophiles/lymphocytes ratio, lactate dehydrogenase, creatinine phosphokinase, aspartate aminotransferase, glucose, cortisol, total plasma proteins, albumin, globulins, albumin/globulins ratio, alkaline phosphatase, glutamate dehydrogenase, IgA, and IgG were determined. Overall, the results of this study indicate that the welfare of pregnant ewes fed with tritordeum meadow was better than that of pregnant ewes fed with oat meadow. Tritordeum meadow had a positive influence on the physiological parameters of animal welfare studied in pregnant Merino ewes. Therefore, tritordeum meadow can be considered a functional feed, as it has a beneficial effect on health beyond its basic nutritional value. Farmers are recommended to feed a cereal such as tritordeum grassland to their sheep, as it not only ensures that the animals benefit from all the nutrients, but also prevents diseases and improves their quality of life. In addition, the cereal’s resistance to fungal diseases makes it suitable for use in sustainable production systems with a reduced environmental footprint.

## 1. Introduction

A prolonged drought is affecting the agricultural and livestock sectors in Spain. Since the beginning of the hydrological year on the first of October 2022 to date, the average rainfall has been 27.5% below the average for this period. The lack of rainfall and the shortage of water in the reservoirs are leading farmers and livestock breeders to an extremely “critical” situation that threatens, imminently, to devastate the crops of many farms in Andalusia, demanding solutions to the sector and a deep reflection of the production model to acquire food demanded by livestock.

This crisis has brought a particularly severe impact on cereal crops, such as oats and wheat, with consequences for animal welfare due to the lack of fodder. In this situation, the inclusion of tritordeum grass as an alternative feed can be a useful option for livestock farmers facing the problem of climate change and rising temperatures, as it is a cereal with yields similar to wheat, but with high resistance to drought and high-temperature stress [1]. Particularly, it is a hardy crop with good disease resistance (*Fusarium culmorum* and *Septoria nodorum*) and low water and fertiliser requirements [2]. These characteristics make it suitable for use in sustainable and low-environmental-impact production systems [3].

Hexaploid tritordeum is the fertile amphiploid (2n = 6x = 42, AABBH chH ch) between wild barley (*Hordeum chilense*) and durum wheat (*Triticum turgidum*). This new species, resulting from the synthesis of hundreds of different amphyloids, has a vast genetic variability available for breeding [4]. Tritordeum’s properties are largely determined by the *H. chilense* genome [5].

A fundamental requirement for the welfare and health of animals lies in nutrition [6,7,8]. Given that nutrition during gestation is, of all the environmental factors, the most important quantitatively in the environment–gene relationship [9,10], and that the foetal period is characterised as a time when the new individual possesses enormous plasticity and capacity to respond to the maternal lifestyle and environment [11], maternal nutrition plays a key role at this stage as it induces permanent DNA methylation [12]. Dietary components can affect gene function and expression in utero and during early life by modulating epigenetic mechanisms mediated by folate metabolism in carbohydrate metabolism or transmethylation processes affecting DNA methylation, histone methylation, or non-coding miRNAs [11]. Questions about what constitutes an appropriate diet to ensure the welfare of pregnant ewes are therefore natural. In intensive livestock production, formulated diets are used, and adequate nutrition is achieved when the nutritional requirements of the average animals in the flock are met [13].

The growing demand for healthier foods, including foods derived from whole grains, has promoted research on health-related traits in human-food tritordeum [14,15,16,17,18,19,20]. However, there is a lack of bibliography dedicated to the use of tritordeum in animal feeds and its effects and repercussions on animal welfare [3,21,22]. The nutritional composition of tritordeum forage in southern Spain coinciding with the meadow production in this study has been described by Salcedo et al. [15].

As mentioned above, tritordeum is well incorporated into human food. Since nutrition ensures the herd’s productive success, it is time to include tritordeum in animal feeds to promote their properties and effects on animal welfare. Traditionally, the most used winter cereal for grazing with sheep is oats, which has a more demanding water requirement than tritordeum [1]. The aim of this study was to compare two different diets (tritordeum meadow vs. oat meadow) for grazing in pregnant ewes to determine whether there was any effect on the objective physiological indicators of animal welfare.

## 2. Materials and Methods

### 2.1. Animals

The research was conducted in an ovine farm located in Pedroche, a village in Valle de los Pedroches (north to the Córdoba province, Spain). This farm had two adjacent meadows of 30 hectares each, one sown with tritordeum and the other with oats. A total of 150 pregnant Merino ewes aged between 3 and 5 years were evaluated. Prior to the start of the study, according to the protocol described by [23], the ewes were synchronised for oestrus using vaginal sponges. After 45 days, 150 pregnant animals were selected by using the MyLabOneVet ultrasound (ESAOTE, Barcelona, Spain) equipped with a 6–10 MHZ multifrequency linear transducer to determine gestation status. From the 60th day of gestation, the first samples were collected to avoid the stress that could be caused by ultrasonography. None of them gave birth during the study, although the study ended in the fifth month of gestation.

All animals were in good health and free from infectious or contagious diseases or parasitic infestations at the beginning of the study. These ewes were randomly divided into two groups: group 1 (n_1_ = 75) was fed with tritordeum meadow and group 2 (n_2_ = 75) with oats meadow during the spring season. Ewes were fed ab libitum on meadow for 12 h. Water was available ab libitum.

### 2.2. Sampling

Blood samples were taken from each ewe at the beginning and at the end of the study. These were used to assess the effect of meadow feeding on parameters related to animal welfare (haematological, metabolic, and immune parameters) and stress (leukocyte parameters, cortisol, and glucose). The cortisol circadian rhythm was considered at the time of the sampling. The hour of extraction was set at 2:00 p.m. after a nap period to avoid interference from the morning cortisol peaks [24]. Three months were left between samplings. The sampling frequency was low to prevent the stress of handling the animals, and its influence on the parameters was studied (i.e., cortisol).

The external jugular vein was punctured to obtain blood samples. Vacutainer^®^ tubes (with and without anticoagulant) were used. The bloods were separated into three aliquots: one in a tube containing EDTA anticoagulant, one in a tube containing heparin-lithium, and one without anticoagulant. The EDTA aliquot was kept refrigerated until further analysis in the laboratory within 24 h of collection. The heparin lithium and anticoagulant-free aliquots were centrifuged at 3000 rpm within 10 min of collection. Plasma and serum were obtained, and both were frozen at −20 °C until further analyses.

### 2.3. Physiological Parameters

The physiological parameters analysed in blood were red blood cell (RBC) count, haemoglobin (Hb), packed cell volume (PCV), and white blood cell (WBC) count. Neutrophils and lymphocytes % were measured, and the neutrophil/lymphocyte ratio was calculated. Parameters analysed in plasma were lactate dehydrogenase (LDH), creatinine phosphokinase (CPK), aspartate amino transferase AST, glutamate dehydrogenase (GDH), total plasma proteins (PPT), and glucose. Parameters investigated in serum were albumin, globulins, alkaline phosphatase (FAL), cortisol, IgA, and IgG.

### 2.4. Assays

A BHA-3000 Veterinary Automatic Haematology Analyser (Getein, Biotech, Inc. Nanjing, China) was used to determine blood parameters (RBCH, Hb, PCV, WBC). On the other hand, LDH, CPK, AST, glucose, and cortisol were determined in serum as described by [25]. For CPK, glucose, cortisol, and LDH, we used an immunoassay analyser (Model Cobas 6000-C501; Roche, Rotkreuz, Switzerland) with commercial kits for CK (kit ref 07190794190, Roche), glucose (kit ref 04404483190), cortisol (kit ref 06687733190), and LDH (kit ref 03004732122). Commercial kits (BioSystems S. A., Costa Brava, Barcelona, Spain) were used for PPT, albumin, globulins, and FAL in serum samples. According to the manufacturer’s instructions, IgA and IgG were determined using ELISA (commercial kits from Euroimmun, Lübeck, Germany). For the determination of GDH enzyme activity, an assay kit #ab10252 (Abcam, Cambridge, UK) was used in accordance with the manufacturer’s instructions. GDH activity was measured as a colour change (λmax = 450 nm) using a PHERAstar Fs (BMG Labtech, Ortenberg, Germany).

### 2.5. Statistical Analysis

Statistical analyses were performed with SPSS v29 (IBM^®^ Statistical Package for the Social Sciences). First, the normality of the data was analysed by means of the Kolmogorov–Smirnov test using the Lilliefors-corrected statistic [26] to determine whether parametric or nonparametric statistics should be applied. Subsequently, the mean and its standard error were calculated for the two groups with different types of feeds, considering both the initial measurement (T0) and the final measurement (T1). Finally, parametric statistical techniques were applied to conclude the analysis and determine the effect of one diet or the other on the means of the parameters analysed. The use of these techniques was also justified because the sample was sufficiently large. Each feeding group constituted a dependent sample, since each animal in it had two measurements of the parameters studied, one initial (T0) and one final (T1). The Student’s *t*-test for paired samples was applied [27]. This test enabled us to determine whether there were statistically significant differences between the initial and final means of each of the variables analysed, with a significance level of 5% for the contrasts. The results are presented in tables grouped in four different blocks of types of parameters related to animal welfare.

## 3. Results

First, the mean and mean standard error were calculated for each parameter in the two feeding groups considered (oats and tritordeum). Subsequently, a contrast of means for dependent samples (paired) was performed in each of them to study the differences between the means of the two measurements of each of the parameters. Table 1, Table 2, Table 3 and Table 4 show the mean and standard deviation errors of the mean of the parameters that make up the group for the two types of feedings, as well as the limiting probability associated with the *t* statistic that enables us to conclude on the contrast of hypotheses, and to see the effect that each diet has on the mean of the parameters analysed.

### 3.1. Haematological Parameters Related to Animal Welfare

Table 1 shows statistically significant differences between the mean values of all haematological parameters related to animal welfare between the initial and final measurements for both oat-fed and tritordeum-fed ewes (*p* < 0.001). Regarding the variation that occurs in each of the parameters analysed, it can be observed how these decrease in the oat-fed group, while they increase in the tritordeum-fed group.

### 3.2. Leukocyte and Stress-Related Parameters

Table 2 shows the results of mean comparison tests for leukocyte and stress-related parameters. It shows that there are no statistically significant differences in the mean values of leukocytes (*p* = 0.343) and neutrophils (*p* = 0.351) in the oat-fed ewes between the two measurements. In contrast, in the tritordeum meadow-fed ewes, differences are detected between the two measurements for all parameters considered in this group (*p* < 0.001).

Focusing attention on lymphocytes, whose mean shows statistically significant differences between the two measurements for both diets, the increase suffered by the animals fed with tritordeum (*p* < 0.001) is notably higher than that of those fed with oats (0.002 *). On the other hand, the neutrophil/lymphocyte ratio, whose mean also shows statistically significant differences for the two diets, shows a much more accentuated decrease in the group fed with tritordeum (*p* < 0.001) (Table 2).

Cortisol, which shows statistically significant differences between the mean of the two measurements for the two feeds (*p* < 0.001), particularly increases for the oat diet while it decreases notably for the tritordeum diet. Finally, the differences detected in both diets in mean glucose are more relevant in the case of the tritordeum diet (Table 2).

### 3.3. Metabolic Parameters Related to Animal Welfare

Regarding the analysis of the differences observed in the metabolic parameters related to animal welfare, it is of particular interest how they vary in the opposite direction depending on whether the diet is oat or tritordeum (Table 3). This is the case for LDH, whose mean values between measurements show statistically significant differences in the two diets, increasing in the case of oatmeal and decreasing significantly in the case of tritordeum; a similar behaviour is observed for the parameters FAL and GDH.

The rest of the parameters that do not present statistically significant differences in the group of sheep fed with oats, do for the diet with tritordeum. Thus, there is a decrease in CPK and AST, and an increase in PPT and albumin.

### 3.4. Immunological Parameters Related to Animal Welfare

In Table 4, the results corresponding to the immunological parameters related to animal welfare show statistically significant differences between the means of the two measurements for the ovine IgA parameters (*p* < 0.001) when focusing on the oat diet. In contrast, statistically significant differences are detected between the means of the initial and final measurements for the four parameters in the animals fed tritordeum (*p* < 0.001).

Particularly for the two diets considered, an increase in ovine IgA is observed between the two measurements. An increase in globulins and IgG is also observed in animals fed tritordeum. In contrast, the mean albumin/globulin decreases between the two measurements.

## 4. Discussion

Understanding a sheep’s metabolic profile, including physiological parameters (blood, plasma, and serum indicators), is essential for its determining nutritional status and preventing health problems that affect production and reproduction [28]. Although the aim of this research is to study the influence of two different diets on animal welfare and not on gestation, it should be pointed out that gestation brings with it a physiological response that influences, for example, the concentration of albumin and total protein with a decrease in their levels due to a rapid extraction of immunoglobulins from plasma in the last months of gestation when colostrum is formed [29]. The increase in protein due to foetal development [30] and the increase in AST enzyme activity show an increase in liver metabolism derived from a decrease in dry matter intake at the end of gestation [31]. The increase in both oxygen demand and metabolic rate contribute to augment the values of red blood cells, haemoglobin, and haematocrit [32,33]. However, the changes obtained in this study do not mean that they are associated with gestation but with feeding; hence, all ewes were chosen at the same physiological state so they would not interfere with the results.

The results indicate that grazing with tritordeum affects haematological parameters and thus animal welfare. The effect of diet was significant; therefore, it should be noted that tritordeum showed an increase in all mean values in the final sampling. These results are not consistent with those obtained in a preliminary study by Requena et al. [22], which compared ewes fed maize silage with those fed tritordeum silage. They observed an increase in haematological parameters in favour of tritordeum silage but did not consider that it affected the welfare of the ewes because this increase was not statistically significant. The lack of agreement with these authors may be due to differences in pH in silage and in grazing. For ewes, the fourth and fifth months of gestation represent the periods with the greatest impact on foetus growth [34]. The red blood cells, haemoglobin, and haematocrit values obtained were higher than those reported in late-pregnancy ewes by Cihan et al. [35], Kalif et al. [36], Habibu. et al. [37], and Yemilmez et al. [38]. These differences may be due to the influence of breeding and nutrition [32,33,35]. The physiological concentrations of the haematological parameters found in these Merino ewes could be associated with their adaptation to the environmental and the agricultural conditions of the Valle de los Pedroches, Cordoba [39].

Elevated cortisol levels were found in ewes fed with oats. This may be due to the incomplete hepatic metabolization of cortisol [40] as well as nutritional and environmental stress triggering the hypothalamic-pituitary-adrenal axis [41]. The hypothalamus-pituitary-adrenal gland axis regulates cortisol expression and contributes to several changes during pregnancy [42]. These changes occur mainly since the increase in cortisol is conditioned by oestrogen regulating plasma corticosteroid-biding globulin levels (corticosteroid binding globulin increases with increasing oestrogen) and placental function [43]. The chronic increase in adrenocorticotropic hormone (ACTH) and cortisol during gestation does not reveal either a relative or absolute opening of the feedback loop between cortisol and hypothalamus and adenohypophysis in pregnant ewes [44].

The authors are aware that blood sampling at 2 pm is unusual, but the management of the farm insisted that samples were taken at that time. Regarding cortisol, no bibliographic references were found to support the time chosen by the authors, but circadian rhythmicity was considered to interpret the results of plasma cortisol [42,45]. It is crucial that this parameter is always sampled at the same time of the day throughout the study to minimise the pulsatile variation of its secretion [24].

During the fifth month of gestation, our results showed that glucose levels increased in both diets. Although glucose levels have been reported to decrease significantly in late pregnant ewes [46,47], some researchers have found high glucose concentrations during this period [48,49], while others have found no significant differences in reproductive glucose [50]. In late pregnancy, glucose is mainly metabolised by tissues that do not depend on insulin for glucose uptake, particularly the foetus. Previous experiments have shown that the foetus accounts for 70% [51] of glucose metabolism in late pregnancy and metabolism in late-pregnancy ruminants. 

Blood glucose is regulated by a complex and an efficient endocrine control that the diet-associated organism keeps over-concentrated, enabling it to remain very constant regardless of diet-associated factors [52]. Nevertheless, tritordeum meadow contributed to a decrease in cortisol levels and an increase in glucose levels. The main source of glucose in ruminants is neoglucogenesis, with a minor contribution from intestinal absorption [53]. The liver plays an important role in regulating blood glucose concentration and its delivery to tissues, being virtually the only organ where neoglucogenesis takes place, although there is some contribution from the kidney [54]. As most of the glucose from the breakdown of dietary carbohydrates is fermented in the rumen [42], ruminants have a chronic glucose deficit. With reduced cortisol, rumen function is not impaired by nutritional stress, and glucose remains elevated [55]. Feeding oat hay as coarse fodder significantly increases the intake, digestibility, and apparent digestibility of acid detergent fibre and neutral detergent fibre in sheep, improving the body’s absorption and utilization of nitrogen. At the same time, feeding with hay diets makes the pH value of sheep rumen fluid within the normal range and promotes the decomposition of carbohydrate and dietary cellulose in the rumen. By improving the rumen environment and increasing the diversity of the microbiota, it also improves apparent digestibility [56].

Ewes fed with oat grass had an inverted neutrophiles/lymphocytes in comparison to ewes fed with tritordeum meadow. Researchers use the neutrophil/lymphocyte ratio as a complementary measure of stress response [57,58], and it is related to the magnitude of the stress process and the concentration of circulating cortisol [59]. As cortisol levels increase during stress, circulating lymphocytes adhere to the endothelial cells that line the walls of blood vessels and migrate from the circulation to other tissues (spleen, bone marrow, lymph nodes, and skin) where they are retained, leading to a decrease in the number of circulating lymphocytes. Likewise, cortisol stimulates the passage of neutrophils from the bone marrow into the blood and inhibits their passage into other compartments, leading to an increase in mature and immature neutrophils in the circulation [60]. These changes ensure that different cell types are directed to the tissues where they are needed during stress [57]. Feeding the sheep tritordeum contributed to the fact that the neutrophiles/lymphocytes ratio was at physiological levels, thus providing evidence of animal welfare.

A few serum biochemical markers may indicate changes in nutritional metabolism and organ function in animals [61]. Stress, besides negatively affecting animal welfare, stimulates a corticotropin-releasing factor to increase intestinal permeability, enabling the passage of lipopolysaccharides and other antigens, and leaving the liver completely exposed and vulnerable to inflammatory processes [62]. The results of this study show a significant decrease in liver enzyme activity (LDH, AST, CPK, and FAL) in ewes fed on tritordeum meadow. This suggests that tritordeum has a protective effect on liver functionality, which contributes to preventing diseases and contributes to an improvement in the welfare of the animals [63].

Humoral immunity includes circulating antibodies or immunoglobulins (Ig). Circulating Ig levels can be reduced by malnutrition, stress, and bacterial, viral, or parasitic infections [64]. IgA and IgG are synthesised by plasma cells derived from B lymphocytes. IgA plays a key role in the formation of the immune barrier of the intestinal mucosa exposed to attacks by external pathogens [65], while IgG is mainly found in blood plasma and interstitial fluid [64]. The immune responses of Merino ewes presented differences between the tritordeum and oat diets. Nutrition is the principal strategy for developing mature immunity [66], influencing the production of Ig [67]. Ig act as membrane receptors on β-lymphocytes and are used by the immune system for the identification and neutralisation of viruses and bacteria [68]. In the present study, the highest concentration of IgA was found in sheep that consumed tritordeum. This would contribute to the prevention of infections and improve their productive performance and antioxidant capacity [68].

Albumin, globulins, and the albumin/globulin ratio are considered nutritional and inflammatory biomarkers. The albumin/globulin ratio is a biochemical parameter used to interpret disease-associated changes in serum proteins. The main clinical use of the albumin/globulin ratio is when it decreases due to decreased serum albumin and increased serum globulins [69]. There were no significant differences at the beginning and at the end of the trial in ewes fed oats meadow. However, a significant increase in both albumin and globulins, presenting a decrease in the albumin/globulin ratio, was observed in ewes fed tritordeum meadow. This finding of an increased concentration of albumin in the plasma is a further indication that these ewes are not exposed to any stress that could impact their welfare and nutritional and immunological statuses [70].

The main limitation of this study was the low frequency of sampling so that the livestock management did not interfere with the results obtained in the physiological parameters evaluated.

In the near future, the authors will investigate the effect of tritordeum on animal welfare by comparing ewes fed tritordeum silage with those fed maize silage and winter cereal silage. They will also evaluate the effect on production levels and animal welfare in lambs at the Cooperativa Ganadera del Valle de los Pedroches (COVAP) typing centre.

## 5. Conclusions

Based on these research results, it can be concluded that feeding tritordeum meadow has a positive influence on the physiological parameters of animal welfare studied in pregnant Merino ewes.

Moreover, tritordeum meadow (i) had a protective effect on liver function (decreased enzyme activity), which contributes to disease prevention; (ii) the increased plasma albumin concentration indicates that these ewes were not exposed to any stresses that could impact their welfare; (iii) nutritional and immune statuses and the increased IgA concentration contributed to the prevention of infections and improved their productive performance and antioxidant capacity. Therefore, tritordeum meadow can be considered a functional feed as it has a beneficial effect on the welfare of pregnant ewes beyond its basic nutritional value.

Tritordeum’s resistance to cereal fungal diseases such as *Fusarium culmorum* and *Septoria nodorum* makes it suitable for use in sustainable production systems with a reduced environmental footprint. It is also suitable for forage, as it has less demanding growing conditions than maize and triticale.

It is recommended that farmers feed their ewes a cereal such as tritordeum, as this not only ensures that the animals use all the nutrients, but also prevents disease and improves their quality of life.

## Figures and Tables

**Table 1:** Haematological parameters related to animal welfare.

	Oat Meadow			Tritordeum Meadow		
Measurements	Initial	Final	*p*-Value	Initial	Final	*p*-Value
	Mean ± SEM	Mean ± SEM		Mean ± SEM	Mean ± SEM	
Red blood cell (10^6^/mm^3^)	9.80 ± 0.07	9.41 ± 0.06	<0.001 *↓	9.93 ± 0.07	10.68 ± 0.07	<0.001 *↑
Haemoglobin (g/dL)	9.89 ± 0.07	9.62 ± 0.06	<0.001 *↓	10.02 ± 0.07	11.46 ± 0.11	<0.001 *↑
Haematocrit (%)	32.71 ± 0.22	31.71 ± 0.21	<0.001 *↓	32.75 ± 0.23	35.28 ± 0.16	<0.001 *↑

Note: Calculation of mean ± SEM and *t*-test for comparison of means for dependent samples. * Statistically significant differences at least at 5% detected in the mean comparison test between the initial and final measurements for each type of feeding. The symbols ↑ and ↓ indicate whether the significant difference detected is an increase or a decrease in the mean at the final measurement.

**Table 2:** Leukocyte and stress-related parameters.

	Oat Meadow			Tritordeum Meadow		
Measurements	Initial	Final	*p*-Value	Initial	Final	*p*-Value
	Mean ± SEM	Mean ± SEM		Mean ± SEM	Mean ± SEM	
Leukocytes (10^3^/mm^3^)	6.61 ± 0.22	6.67 ± 0.21	0.343	6.49 ± 0.21	7.14 ± 0.18	<0.001 *↑
Neutrophiles (%)	47.53 ± 0.825	47.16 ± 0.562	0.351	48.52 ± 0.784	42.81 ± 0.61	<0.001 *↓
Lymphocytes (%)	42.83 ± 0.65	43.96 ± 0.41	0.002 *↑	43.23 ± 0.75	53.95 ± 0.78	<0.001 *↑
N/L	1.14 ± 0.03	1.08 ± 0.01	<0.001 *↓	1.14 ± 0.02	0.81 ± 0.02	<0.001) *↓
Cortisol (*μ*g/dL)	11.81 ± 0.47	12.03 ± 0.42	0.048 *↑	12.01 ± 0.47	7.41 ± 0.31	<0.001 *↓
Glucose (mg/dL)	64.55 ± 1.55	73.09 ± 1.16	0.001 *↑	63.71 ± 1.34	84.75 ± 1.42	<0.001 *↑

Note: Calculation of mean ± SEM and t-test for comparison of means for dependent samples. * Statistically significant differences at least at 5% detected in the mean comparison test between the initial and final measurements for each type of feeding. The symbols ↑ and ↓ indicate whether the significant difference detected is an increase or a decrease in the mean at the final measurement.

**Table 3:** Metabolic parameters related to animal welfare.

	Oat Meadow			Tritordeum Meadow		
Measurements	Initial	Final	*p*-Value	Initial	Final	*p*-Value
	Mean ± SEM	Mean ± SEM		Mean ± SEM	Mean ± SEM	
LDH (IU/L)	641.15 ± 8.01	664.45 ± 7.11	<0.001 *↑	640.16 ± 8.217	536.21 ± 13.482	<0.001 *↓
CPK (IU/L)	379.41 ± 18.68	369.37 ± 16.85	0.546	387.57 ± 18.80	286.27 ± 17.59	<0.001 *↓
AST (IU/)	128.60 ± 2.34	125.85 ± 1.71	0.344	130.28 ± 2.087	111.95 ± 3.806	<0.001 *↓
FAL (IU/L)	114.68 ± 5.91	133.28 ± 5.584	<0.001 *↑	115.15 ± 5.91	90.64 ± 2.71	<0.001 *↓
PPT (g/dL)	6.97 ± 0.04	6.95 ± 0.04	0.766	6.96 ± 0.04	7.27 ± 0.03	<0.001 *↑
GHD (IU/L)	9.29 ± 0.36	9.35 ± 0.36	<0.001 *↑	8.69 ± 0.32	7.26 ± 0.27	<0.001 *↓
Albumin (g/dL)	3.16 ± 0.02	3.16 ± 0.02	0.906	3.14 ± 0.03	3.17 ± 0.02	<0.001 *↑

Note: Calculation of mean ± SEM and *t*-test for comparison of means for dependent samples. * Statistically significant differences at least at 5% detected in the mean comparison test between the initial and final measurements for each type of feeding. The symbols ↑ and ↓ indicate whether the significant difference detected is an increase or a decrease in the mean at the final measurement.

**Table 4:** Immunological parameters related to animal welfare.

	Oat Meadow			Tritordeum Meadow		
Measurements	Initial	Final	*p*-Value	Initial	Final	*p*-Value
	Mean ± SEM	Mean ± SEM		Mean ± SEM	Mean ± SEM	
Globulin (g/dL)	3.78 ± 0.048	3.67 ± 0.05	0.080	3.66 ± 0.06	4.03 ± 0.04	<0.001 *↑
Albumin/globulin	0.84 ± 0.01	0.87 ± 0.01	0.101	0.87 ± 0.01	0.79 ± 0.01	<0.001 *↓
IgA (mg/dL)	7.54 ± 0.23	7.76 ± 0.23	<0.001 *↑	7.34 ± 0.24	9.13 ± 0.30	<0.001 *↑
IgG (mg/dL)	633.85 ± 21.65	655.65 ± 21.718	0.118	634.57 ± 20.65	820.58 ± 31.98	<0.001 *↑

Note: Calculation of mean ± SEM and *t*-test for comparison of means for dependent samples. * Statistically significant differences at least at 5% detected in the mean comparison test between the initial and final measurements for each type of feeding. The symbols ↑ and ↓ indicate whether the significant difference detected is an increase or a decrease in the mean at the final measurement.

## Data Availability

The data presented in this research are presently unavailable for being part of an unfinished doctoral thesis.
